# Hook plate fixation of Neer type II distal clavicle fractures results in satisfactory patient-reported outcomes but complications and revisions are high

**DOI:** 10.1186/s12891-023-06975-w

**Published:** 2023-10-27

**Authors:** Nils Beisemann, Yannik M. Spiller, Marc Schnetzke, Paul Alfred Grützner, Philip-Christian Nolte

**Affiliations:** 1 Department for Trauma and Orthopedic Surgery, BG Klinik Ludwigshafen, Ludwigshafen am Rhein, Germany; 2 German Joint Centre, ATOS Clinic Heidelberg, Heidelberg, Germany

**Keywords:** Distal clavicle fracture, Hook plate, Neer type II clavicle fracture, Retrospective study

## Abstract

**Background:**

Surgical treatment of distal clavicle fractures Neer type II is challenging. A gold standard has not yet been established, thus various surgical procedures have been described. The purpose of this study is to report the radiological and clinical outcomes using hook plate fixation in Neer type II distal clavicle fractures.

**Methods:**

We retrospectively reviewed data of 53 patients who underwent hook plate fixation between December 2009 and December 2019 with ≥ 2 years of follow-up. Patients with preexisting pathologies or concomitant injuries of the ipsilateral shoulder were excluded. Pre- and postoperative coracoclavicular distance (CCD), bony union and patient-reported outcomes were collected, including the Constant Score (CS) and Subjective Shoulder Value (SSV). Complications and revisions were recorded.

**Results:**

At a mean final follow-up of 6.2 years, mean SSV was 91.0% (range, 20–100) and mean CS was 80.9 points (range, 25–99). The mean preoperative CCD was 19.0 mm (range, 5.7–31.8), the mean postoperative CCD was 8.2 mm (range, 4.4–12.2) and the mean CCD following hardware removal was 9.7 mm (range, 4.7–18.8). The loss of reduction following hardware removal was statistically significant (P = 0.007). Eleven (20.8%) patients had complications, with 5 cases of deep or superficial infection (9.4%), four non-unions (7.5%), one periosteosynthetic fracture, one postoperative seroma, one implant failure and one symptomatic acromioclavicular joint arthritis (all 1.9%). A total of 10 patients (18.9%) underwent revision surgery at a mean of 113 (range, 7–631) days.

**Conclusion:**

Medium-term patient-reported outcomes for hook plate fixation of Neer type II distal clavicle fractures are satisfactory; however, one in five patients suffers a complication with the majority of them requiring revision surgery.

## Background

The treatment of distal clavicle fractures largely depends on fracture morphology. Whereas clavicle fractures Neer type I and III are successfully treated nonoperatively, type II fractures are considered unstable due to compromised coracoclavicular (CC) ligaments and therefore do not respond well to nonoperative treatment with non-union rates of up to 33% [1, 2].

Despite the need for surgical care, a gold standard for the treatment of Neer type II fractures has not yet been established and optimal treatment remains controversial. Numerous procedures such as osteosynthesis with anatomically precontoured plates [3], hook plates [4, 5], intramedullary screws, temporary K-wire transfixation [6] as well as ligament bracing [7] and the combination of ligament bracing and plate osteosynthesis have been described [8].

Hook plates are commonly used because their design combines two advantages: the distal fracture fragment can be fixed with screws allowing for osteosynthesis and dislocating forces are neutralized due to the hook that is placed under the acromion allowing for stabilization of the acromioclavicular joint.

However, in open reduction and plate fixation of distal clavicle fractures wound complications are not uncommon [9]. Additionally, subacromial erosions and rotator cuff injury have been reported with the use of hook plates [9] and thus, removal is generally recommended resulting in a second surgery.

Therefore, some authors avoid open reduction and plate fixation and advocate for less invasive methods such as internal bracing or a combination of internal bracing and CC ligament reconstruction [10, 11]. However, in a meta-analysis Panagopoulus et al. reported the highest complication rate of 46% with the minimally-invasive procedure of AC joint transfixation, followed by hook plates with 42% and locking plate fixation of 23.6%. [2].

Therefore, the purposes of this study were to report patient-reported and radiographic outcomes and to assess complications and revisions using hook plate fixation in Neer type II distal clavicle fractures.

## Methods

This retrospective, single-center study was performed at a level 1 trauma center. Patients who underwent hook plate fixation between December 2009 and December 2019 for Neer type II distal clavicle fractures within the first 3 weeks following trauma and were at least 2 years out from surgery were eligible for inclusion. Exclusion criteria were: preexisting pathologies or concomitant injuries about the ipsilateral shoulder, an age of less than 18 years at the time of injury, severe language barrier, incarceration or serious disability precluding them from participation. A total of 74 patients met the inclusion criteria. Of those, two had concomitant injuries about the ipsilateral upper extremity, one had a severe language barrier, two were incarcerated at time of injury and/or follow-up and three had se-rious disability or illness precluding them from participation in the study. This left a total of 66 patients for inclusion.

### Surgical management

Following general anesthesia patients were placed in the beach chair position. All included patients underwent hook plate fixation (Synthes, Umkirch, Germany) according to the principles of the AO Foundation (Arbeitsgemeinschaft für Osteosynthesefragen, Davos, Switzerland). Length of the plate and depth of the hook was chosen based on the individual anatomy of the patient. Following reduction of the fracture, at least three bicortical screws were placed proximal to the fracture. Depending on bone quality up to two locking screws were used in the proximal fragment. If fracture morphology allowed for screw placement in the distal fragment this was performed as well using either locking or non-locking screws depending on the bone quality.

### Postoperative rehabilitation

The treated extremity was placed in a shoulder sling until swelling subsided and the wound has had healed. Passive and active-assisted abduction and flexion of the shoulder to 90 degrees was performed four weeks postoperatively and started on the second postoperative day. Starting from the fifth postoperative week, active abduction and flexion to 90 degrees was allowed. Hook plate removal was recommended four months postoperatively. Until the plate was removed, abduction and flexion over 90 degrees was not recommended to avoid subacromial erosion or rotator cuff injury. Following hook plate removal, full active range of motion was allowed.

### Demographic data and Patient-reported outcome scores

Demographic data were assessed using patient medical records and included age, sex, injury mechanism, time from injury to surgery, length of stay for initial surgery and time from initial surgery to hardware removal. At minimum 2-year follow-up, patient-reported outcome scores that were collected included the Constant score (CS) [12] and the Subjective Shoulder Value (SSV) [13]. In addition, patients completed a questionnaire regarding hardware removal and time to hardware removal if performed in another hospital and preexisting conditions/previous surgery of the treated shoulder joint. Complications and revision surgeries were assessed.

### Radiographic assessment

Radiographs were not routinely performed for study purposes; however, standard anteroposterior radiographs were available in all patients preoperatively allowing for fracture classification and measurement of the coracoclavicular distance (CCD). Patients with available postoperative radiographs following hook plate fixation and radiographs following hook plate removal were assessed for bony union and CCD. CCD [14] was measured from the superior aspect of the coracoid to the inferior cortex of the clavicle. [15–17] Bony healing was assessed with the latest available radiographs and defined as invisible fracture line or bridging callus across the fracture line.

### Statistical analysis

Normality of data was assessed using the Shapiro Wilk test. Continuous data were reported as mean and range with 95% confidence intervals. Parametric, continuous data were analyzed using the dependent samples t-test and nonparametric data using the Mann-Whitney U test. To assess for statistical significance two-tailed P values were calculated, and significance was set at P < 0.05. Statistical analysis was performed using Prism software (GraphPad, version 9.3.1, San Diego, CA, USA).

## Results

**Fig. 1:**
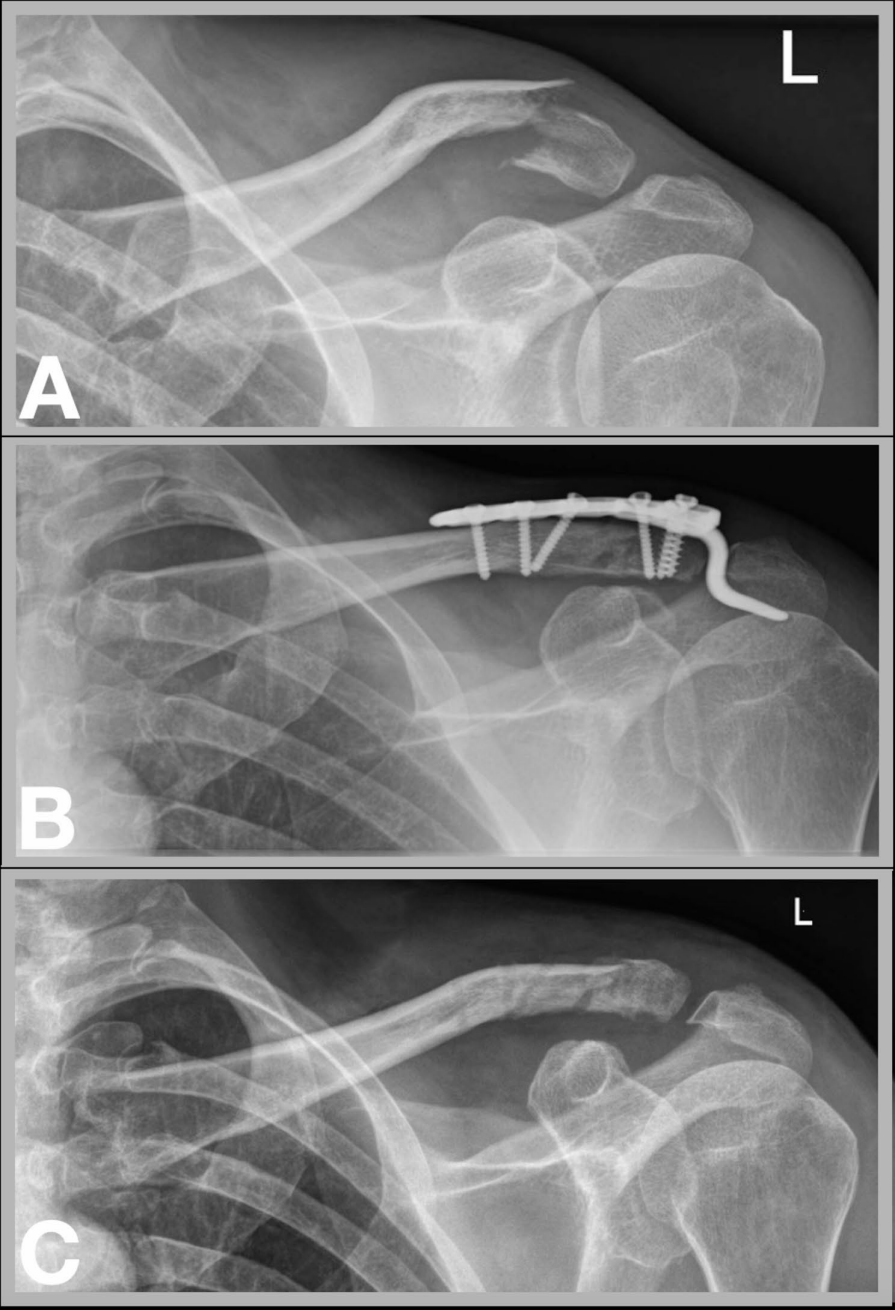
Treatment of a distal clavicle fracture with a hook plate and subsequent elective hardware removal. (**A**) Preoperative radiograph of a Neer type II distal clavicle fracture, (**B**) Fracture treatment with a hook plate, (**C**) Postoperative radiograph after elective hardware removal

Out of 66 patients, a total of 53 patients (80.3%) were available for follow-up at a mean of 6.2 (range, 2–10) years. There were 40 (75.5%) men and 15 (24.5%) women with a mean age of 51.0 (range, 18–81) years. Injury mechanisms were bicycle accidents in 22 (41.5%), falls from standing height in 11 (20.8%), motorcycle accidents in 8 (15.1%), horseback riding accidents in 5 (9.4%) and other type of accidents in 7 (13.2%) patients. Mean duration from injury to operative treatment was 6.8 (range, 4–16; 95% CI, 5.8–7.7) days and mean length of stay was 7.1 (range, 2–24; 95% CI, 5.6–8.7) days. All patients (100%) underwent hook plate removal after a mean of 113 (range, 45–529; 95% CI, 90–137) days, which equals 16.1 weeks. Figure 1 shows the treatment of a distal clavicle fracture with a hook plate and subsequent elective hardware removal.

At final follow-up, mean SSV was 91.0% (range, 20–100; 95% CI, 86.8–95.1) and mean CS was 80.9 points (range, 25–99; 95% CI, 75.1–86.0).

**Fig. 2:**
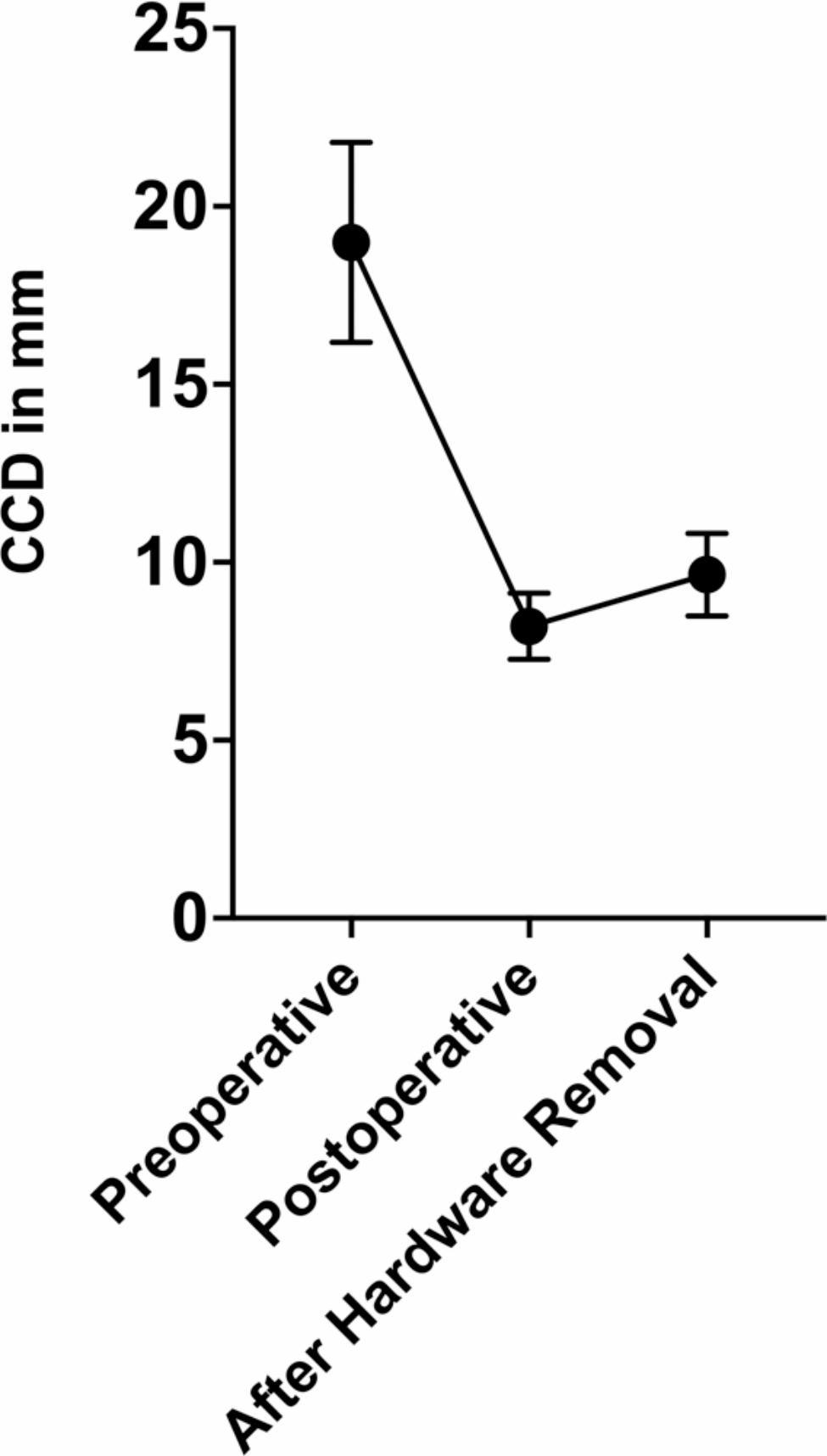
Graph demonstrating the change in CCD at pertinent times during hook plate treatment. CCD: coracoclavicular distance

Complete radiographic data, including preoperative and postoperative radiographs as well as radiographs after hardware removal, were available for 28 (52.8%) patients. The mean preoperative CCD was 19.0 mm (range, 5.7–31.8; 95% CI, 16.2–21.8), the mean postoperative CCD was 8.2 mm (range, 4.4–12.2; 95% CI, 7.3–9.1) and the mean CCD following hardware removal was 9.7 mm (range, 4.7–18.8; 95% CI, 8.5–10.8). There was a statistically significant loss of reduction as measured by difference in CCD between postoperative radiographs (8.2 mm [range, 4.4–12.2; 95% CI, 7.3–9.1]) and radiographs performed after hardware removal (9.7 mm [range, 4.7–18.8; 95% CI, 8.5–10.8]; P = 0.007) (Fig. 2).

**Fig. 3:**
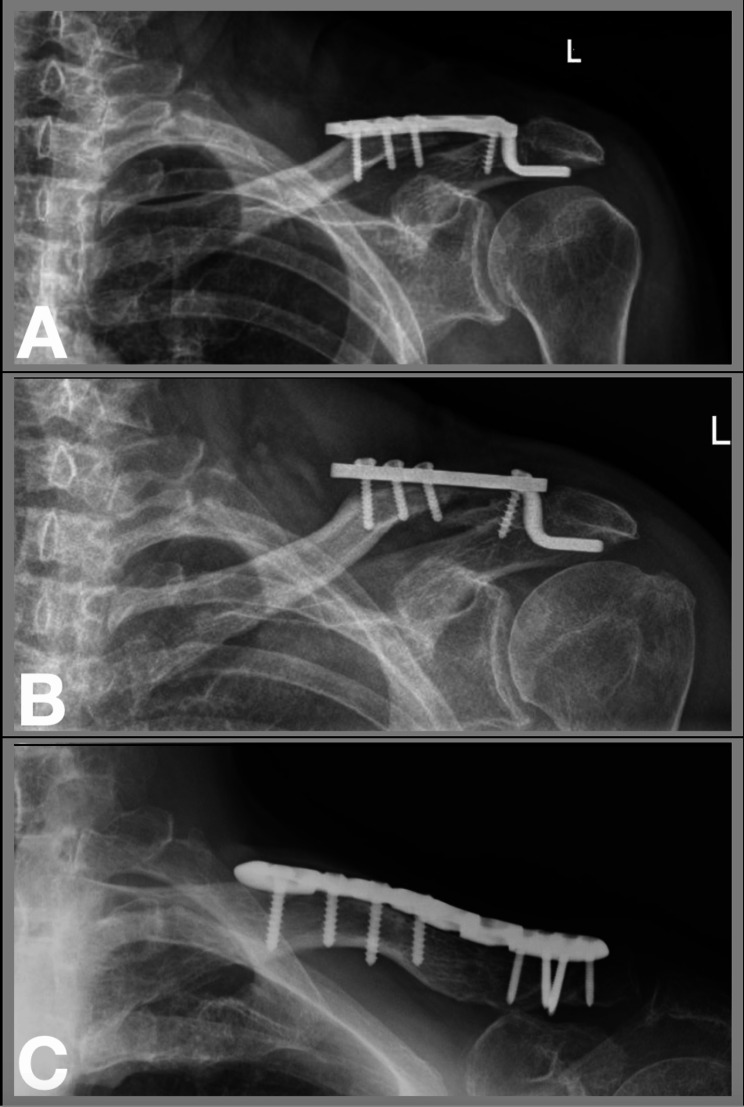
Case example of a 64-year-old female with non-union and subsequent re-osteosynthesis using an anatomical locking plate. (**A**) Postoperative radiograph following hook-plate, (**B**) Non-union and implant failure, (**C**) Radiograph demonstrating bone union follwoing re-osteosynthesis with an anatomical locking plate and autologous iliac crest graft

Out of 53 patients, a total of 11 (20.8%) patients had complications. The most common complication was deep or superficial infection (5 patients [9.4%]), followed by non-union (4 patients [7.5%]), periosteosynthetic clavicle fracture, postoperative seroma, implant failure and symptomatic AC joint arthritis (1 patient each [1.9%]). All patients with a complication but one (periosteosynthetic fracture, which was treated conservatively) underwent revision surgery (10 patients [18.9%]) at a mean of 113 (range, 7–631; 95% CI, -8.1–234.7) days. Details of complications and revision surgeries are listed in Table 1. Figure 3 shows a case of non-union with subsequent reosteosynthesis using a locking plate.

**Table 1:** **Complications and revision surgeries for the total cohort.** AC: acromioclavicular. I&D: irrigation and debridement. SSV: Subjective Shoulder Value

Sex	Age, y	Comorbidity	Complication	Revision	Days to revision	Follow-up, y	SSV, %
Male	30	-	Infection, Non-union	I&D	7	9	95
Male	48	-	Infection	2x I&D	18	4	90
Male	55	-	Infection	I&D	14	9	90
Female	55	-	Peri-osteosythetic fracture	Nonoperative	15	5	60
Male	57	-	AC-Arthritis	Distal clavicle resection	631	7	70
Male	62	High Blood Pressure	Seroma	Debridement and implant removal	45	9	100
Male	63	-	Infection	I&D and implant removal	77	9	70
Female	64	Epilepsy	Non-union	Re-osteosynthesis with autologeous iliac crest graft	161	5	100
Male	71	High Blood Pressure	Infection, Non-union	I&D	16	9	95
Female	76	High Blood Pressure	Non-union	Re-osteosynthesis	118	7	100
Female	80	Nicotine abuse	Implant failure	Implant removal and distal clavicle resection	144	8	20

Although patients with complications had lower patient-reported outcome scores compared to patients without complications, there were no statistically significant differences in mean SSV (80.9% [range, 20–100; 95% CI, 64.4–97.4] vs. 93.5% [range, 40–100; 95% CI, 90.3–96.6]; P = 0.106) and CS (73.5 [range, 37–94; 95% CI, 56.4–90.6] vs. 81.9 [range, 25–99; 95% CI, 76.8–87.0]; P = 0.347).

However, patients of 55 years and older had a significantly lower mean SSV (85.7 [95%CI: 78.3–93.1] vs. 96.9 [95%CI: 95.2 vs. 98.5]; P = 0.004) and mean CS (75.6 [95%CI: 67.4–83.8] vs. 88.0 [95%CI: 84.5–91.5]; P = 0.019) compared to patients younger than 55 years of age.

## Discussion

The most important finding of this study was that hook plate fixation for distal clavicle fractures Neer type II resulted in satisfactory patient-reported outcome scores as demonstrated by a mean SSV of 91.0% and a mean CS of 80.9 points at a median follow-up of 6.2 years. However, despite satisfactory clinical results, complications were unacceptably high (20.8%) with the majority of them requiring revision surgery (18.9%).

Compared with the literature on hook plate fixation, the complication rate in the present study is similar; however, it is striking that the revision rate is higher. Erdle et al. demonstrated a complication rate of 63.2%, but only one case (5.2%) required revision surgery (periimplant fracture) [18]. The other complications comprised of non-unions, delayed unions, acromial osteolyses, and posttraumatic ACJ arthroses [18]. Uittenbogaard et al. performed a meta-analysis investigating 2284 Neer type II fractures and found a complication rate of 24% for hook plate fixation. Again only 4% of the included patients underwent revision surgery [1]. Teimouri et al. compared hook plate versus t-plate in treatment of Neer type II distal clavicle fractures. Here, a revision rate of 6.7% is reported in the hook plate group [19]. In a study by Li et al., no revision was necessary in any of the 81 patients followed up [20].

In another meta-analysis of surgical treatment of Neer type IIb fractures, in the subgroup of hook plate fixation, an even higher overall complication rate was found with 42% of them graded as minor complications and 4.5% graded as major complications; however, the revision rate was not specified [2]. Only Flinkillä et al. reported a revision rate of 16.7%, which is similar to our study [21]. The high revision rate in the present cohort, when compared to the literature, is not fully comprehensible. A possible reason may be an aggressive approach to treat superficial infections or non-union by means of revision surgery in our practice. Non-union occurs in 31% of patients following nonoperative treatment and has been shown to be only mildly symptomatic [2]. Since there was no control group no definitive conclusions on the performance of hook plate fixation can be drawn. However, the high complication and revision rate should alert surgeons.

In the systematic reviews performed by Panagopoulos et al. and Uittenbogaard et al., the authors summarize that the surgical treatment of Neer type II fractures has a high complication rate in general, but the use of hook plates leads to poorer clinical outcomes compared to other modes of fixation [1, 2]. In the present study, a mean SSV of 91.0 was achieved, which is comparable to the literature [22–24], and does not fully agree with the poor results of Uittenbogaard and Panagopoulos [1, 2]. Interestingly, the mean CS of 80.9 is below of what is described in the literature for locking plate fixation, AC Joint transfixation, tension band wiring and hook plate fixation as well [2].

In their meta-analysis, Panagopoulos et al. demonstrated that all compared groups including AC Joint transfixation (CS: 94.3), CC stabilization (CS: 93.8), locking plate (CS: 93.1) and hook plate fixation (CS: 87.4) yielded higher Constant Scores than what was shown in the present study [2].

Uittenbogaard et al. came to the conclusion that CC stabilization had lower complications and a significantly higher CS when compared to hook plate fixation [1]. Nevertheless, when comparing hook plate fixation with locking plate fixation and tension band wire/K-Wire fixation, no difference was shown. Furthermore, the union rates were similiar across all operative treatment modalities [1].

However, it is questionable whether the measured difference in CS in our study compared to the investigations discussed before has clinical significance. Kukkonen et al. were able to demonstrate, that in patients who underwent rotator cuff repair the minimally important clinical difference (MCID) in CS is 10.4 points [25]. Differences below that threshold are likely not clinically detectable. Unfortunately, clinical significance values are not yet available for distal clavicle fractures.

Another interesting point is the gender and age specific CS: Balcells-Diaz et al. could show that in healthy population the CS differs with respect to age and gender [26]. Furthermore, Tavakkolizadeh et al. could show that in men from 50 years to 70 years the CS decreases by 0.15 points and 1.3 points above 70 years, and in women between 50 and 70 years it decreases by 0.25 points and above 70 years by 0.35 points [27]. Our study supports this: Patients of 55 years and older had a significantly lower mean SSV (85.7 vs. 96.9; P = 0.004) and mean CS (75.6 vs. 88.0; P = 0.019) compared to patients younger than 55 years of age.

Although no statistically significant difference in SSV and CS between patients with complication and without complication (SSV 80.9% vs. 93.5%, P = 0.106; CS 73.5 vs. 81.9, P = 0.347) a trend is clearly apparent. To our knowledge, there is no study that has clinically compared patients after hook plate fixation with and without complications. However, the high complications rate could explain the generally worse CS for hook plate fixation compared to CC stabilization described by Uittenbogaard et al. [1]. Furthermore, it is conceivable that the patients who needed revision were more dissatisfied with the overall treatment, which is reflected in the worse subjective SSV, than the more objective CS showed.

Although a statistically significant difference in CCD was shown from postoperative CCD to CCD following hook plate removal (8.2 mm vs. 9.7 mm; P = 0.007), the final CCD is still within the physiological norm [22, 28]. Therefore, it can be assumed that the slight increase in CCD does not affect clinical outcomes. Overall, these findings are supported by other groups who demonstrated comparable increases in CCD after hardware removal [29, 30]. The treatment of distal clavicle fractures remains challenging. The data show a high complication and revision rate, so that the surgical indication for hook plates should be very cautious. Fracture types that are amenable to less invasive operative techniques should be treated with such, thereby potentially reducing the risk of infection.

Our study has several limitations. First, this is a retrospective study which renders it prone to bias and loss of data. Complete radiographic datasets were available for only 28 (52.8%) patients, although we achieved a sufficient clinical follow-up rate of 80.3%.

Second, this is a single center study which reduces variability in approach and technique; however, surgeries were performed by or under the supervision of various board certified surgeons. Finally, we only investigated hook plate fixation without using a control group and therefore cannot draw definitive conclusions on the performance of this treatment modality in comparison to other techniques.

## Conclusions

Medium-term patient-reported outcomes for hook plate fixation of Neer type II distal clavicle fractures are satisfactory; however, one in five patients suffers a complication with the majority of them requiring revision surgery.

## Data Availability

The datasets used and analysed during the current study are available from the corresponding author on reasonable request.

## List of Abbrevations

CCD: Coracoclavicular distance
CS: Constant Score
SSV: Subjective Shoulder Value
CC: Coracoclavicular
AC: Acromioclavicular

## Declarations

All patients gave their informed consent for inclusion before they participated in the study.
